# A qualitative assessment of adolescent perspectives on patient education in the outpatient setting

**DOI:** 10.1016/j.pecinn.2022.100117

**Published:** 2022-12-09

**Authors:** Nicole Meyers, Michelle Kaminski, Samuel Master, Marina Catallozzi, Suzanne Friedman

**Affiliations:** a Department of Pediatrics, NewYork-Presbyterian Morgan Stanley Children’s Hospital, 630 W 168th Street, PH 5, East Room 520, New York, NY 10032, USA; b Columbia University Mailman School of Public Health, 722 W 168th Street, New York, NY 10032, USA; c Department of Pediatrics, Columbia University Irving Medical Center, 630 W 168th Street, New York, NY 10032, USA

**Keywords:** Adolescent health, Patient engagement, Patient-centered care, Anticipatory guidance, After-visit summaries, Qualitative research

## Abstract

**Objective:**

To explore adolescent perspectives on the content and delivery of anticipatory guidance (AG), both during and after outpatient visits, in order to develop targeted resources and educational material for adolescent patients.

**Methods:**

Semi-structured phone interviews among patients ages 12 to 21 seen between May-July 2021 at four outpatient sites of NewYork Presbyterian Hospital were recorded, transcribed and analyzed using thematic analysis. Content domains included attitudes toward and preferences around AG, discharge instructions and patient education resources.

**Results:**

Twenty-eight of 156 recruited patients completed interviews; 52% received an After Visit Summary (AVS); of the 48% who did not receive it, half of them expressed interest in receiving one. Themes included positive perceptions of the AVS, patient-physician communication, multimodal delivery of educational materials, and critical discussion topics such as mental health and nutrition.

**Conclusion:**

Adolescents value the AVS and prefer multimodal materials and topics that are specifically geared towards them, rather than their caregivers.

**Innovation:**

This study is the first to explore adolescent perspectives on AG and after-visit informational materials. These findings may help more effectively reach, educate and engage adolescent patients in the primary care setting by guiding the focused development of patient-centered instructions and resources.

## Highlights

• Adolescents perceive patient education material e.g. clinical summaries favorably.
• They prefer counseling material via multiple channels specifically targeted to them.
• The most important topics adolescents wanted to discuss were mental health and nutrition.
• Adolescent perspectives can guide the development of novel educational resources.

## 1 Introduction

National pediatric organizations recommend annual adolescent outpatient visits for necessary screening, counseling and promotion of healthy behaviors [1,2]. However, studies estimate only 38% of adolescents receive their annual preventative visit, the lowest rate of primary care use among all age groups in the United States [3]. Comprehensive anticipatory guidance (AG), which is timely, relevant, and developmentally-appropriate advice around emerging issues the patient and family are likely to face, is infrequently delivered to adolescents during preventative visits [[2], [3], [4], [5], [6], [7], [8], [9]]. Providers must develop efficient and patient-centered ways of delivering prioritized information to improve adolescent engagement with the healthcare system, as health services for adolescents are poorly suited to meet their needs [5,6,10,11]. Numerous barriers to obtaining regular care exist, including adolescents’ lack of interest in and knowledge about the necessity and benefit of these preventative visits [12].

The conclusion of outpatient visits is an opportunity for providers to summarize important takeaways and share information and resources that can be referenced by patients after the visit. The After-Visit Summary (AVS) is a tool through the electronic medical record that provides relevant and actionable take-home information, including diagnoses, medications, future appointments, and often patient-specific anticipatory guidance. The AVS has been proven to enhance patient engagement, and retention and comprehension of information from the visit in certain populations [[13], [14], [15]]. Based on this, the Centers for Medicaid Services recommend provision of an AVS following all healthcare encounters [16].

Adolescents' attitudes toward, perceived utility of, and engagement with the AVS is largely unknown, as previous studies have focused on older patient populations [13,14,17]. Overall, research regarding the delivery of AG both during and after adolescent wellness visits is limited [5,6]. Some studies have assessed adolescent and parent preferences for topics they would like to discuss at clinic visits [18,19], but there is a paucity of research exploring perspectives on provision of AG and health information.

Our study sought to explore adolescent perspectives on the content and delivery of AG, both during and after outpatient visits, in order to develop targeted resources and educational material for adolescent patients.

## 2 Methods

Between May-June 2021, adolescents between the ages of 12 to 21 who were at any of the four NewYork Presbyterian community-based Ambulatory Care Network (ACN) practices in Northern Manhattan were recruited. Adolescents seen for annual well or problem-based follow up visits were included, while those seen for urgent or nurse-only visits were excluded. These ACN practices serve approximately 20,000 pediatric patients annually who are largely publicly-insured (∼71%) and with low-income (∼24% with <$15,000 annual household income) [20].

This study was approved by Columbia University Medical Center’s IRB.

Participation was voluntary, and eligible participants and parents were mailed a study information sheet with the research team’s contact information to opt out if desired. Semi-structured patient phone interviews were conducted by one member of the study team (MK) for patients that agreed to participate. MK requested to speak to the adolescents directly and confirmed they were in a private space, as to ensure no parental presence.

Self-determination theory guided the development of the semi-structured interview guide [21]. Interview guide topics focused on the concepts of adolescent autonomy, competence (assessed by preferred educational topics), and relatedness (with adolescent perceptions of physician communication as a proxy).

After initial development of the interview guide, the study team made iterative revisions to increase clarity and focus on concepts. The guide was finalized after review by both adolescent medicine specialists and general pediatricians to ensure expert consensus. Final interview domains included attitudes toward and preferences around the content and delivery of AG, which included education provided during the visit and resources e.g. the AVS utilized post-visit. Audio interview recordings were transcribed by two study team members (NM & MK).

Transcribed and deidentified interviews were uploaded to the NVivo software platform (Version 12). Using thematic analysis [22], the team identified patterns and two primary analysts developed a code book and applied codes. Disagreement was resolved through review and discussion, resulting in high inter-coder agreement (99.7%) and moderate inter-coder reliability (k=0.45). Themes were identified and interpreted for understanding.

## 3 Results

Of 156 eligible patients from the four practices, 28 patients (17.9%) were successfully interviewed, 65 (41.6%) did not answer and 63 (40.4%) declined. The majority of participants were English-speaking (92.9%), Hispanic (78.5%), and female (64.3%) (Table 1). The median interview length was 6.2 minutes, ranging from 4.9 to 15.2 minutes. Many patients (52%) reported receiving an AVS, and half of those who did not receive one responded they would have liked to.

**Table 1:** Demographics of patients interviewed.

Demographic	Count (Percentage)
Sex Assigned at Birth	
Female	18 (64.3%)
Male	10 (35.7%)
Preferred Language	
English	26 (92.9%)
Spanish	2 (7.1%)
Ethnicity	
Hispanic	22 (78.5%)
Non-Hispanic	3 (10.7%)
Declined	3 (10.7%)
Sites	
Site 1	5 (17.9%)
Site 2	9 (32.1%)
Site 3	5 (17.9%)
Site 4	9 (32.1%)
Age	
Mean (SD)	15 (2.2)

The main themes identified were around the content and delivery of anticipatory guidance, and more specifically included perceptions of the AVS, patient-physician communication, patient education methods and critical discussion topics (Table 2). Sub-themes within perceptions of the AVS were benefits and disadvantages of the AVS including adolescents’ perceptions on whether or not the AVS would help their understanding of their health. Those within patient-physician communication included comprehensive visits, physician communication, and adolescent autonomy. These sub-themes detailed adolescent perspectives on communication with providers during the visit and whether it was targeted toward them or their caregivers. Patient education methods encompassed adolescents’ preferences to learn using electronic resources, paper resources or their own resources both during and after their visits. The final theme of critical discussion topics included anticipatory guidance content that adolescents desired to discuss, ranging from mental health and nutrition to administrative needs such as school forms.

**Table 2:** Qualitative themes, sub-themes and representative quotes.

Theme	Sub-Theme	Representative Quote (Age, Sex Assigned at Birth)
Perceptions of the AVS *(Respondents described their perspective on the AVS)*	Benefits of the AVS *(Respondents described a positive about the AVS)*	*I would have to say it is pretty helpful because some things I wouldn’t understand like what kind of shots I got or stuff like that so being given the description of each, what it is and what it does, it was pretty helpful. (19, Female)*
	Disadvantages of the AVS *(Respondents described a lack of perceived benefit of the AVS)*	*That wasn’t really important to me and it still isn’t important to me because I trust my doctor and I wouldn’t need a summary of our appointment because I wouldn’t look back on it. (15, Female)*
Patient-Physician Communication *(Respondents commented on their communication with their provider during the visit itself)*	Comprehensive Visits *(Respondents report that all their questions were answered and they had no additional issues to discuss)*	*Honestly, she discussed everything I wanted to discuss with her so there is really nothing that she left out. (13, Female)*
	Physician Communication *(Respondents described a personality trait of their physician or aspect of the communication at the visit)*	*She was very easygoing and very talkative and it was nice to have someone. She was very young so it was kind of good to talk to someone kind of young so it was good. (19, Female)*
	Adolescent Autonomy *(Respondents reported that they were the one addressed during their visit)*	*Yeah I feel she was talking more to me. I am 19 and I have to take care of myself. (19, Female)*
Patient Education Methods *(Respondents described sources of education and anticipatory guidance during and after their visits)*	Preference for Electronic Resources *(Respondents expressed preference for electronic resources)*	*I like MyChart, it is very easy to use. Very self-explanatory and just keeps everything in one concise area instead of having things spread out through paper and stuff. (18, Female)*
	Preference for Paper Resources *(Respondents expressed preference for paper resources)*	*I would like a paper with the information of any health events or any other information about my disease that I have. (15, Female)*
	Patients’ Own Resources *(Respondents expressed preference for finding their own sources of information)*	*Well I do my own medical research, I do. I remember the name of my disease and I google it on google and then there are specific types of medicines or treatments that I would read about and then I would ask my doctor about them and which ones she would suggest I do. (15, Female)*
Critical Discussion Topics *(Respondents described priority anticipatory guidance topics discussed or to be discussed)*	Mental Health *(Respondents described a preference for discussions about mental health with their providers)*	*She gave me some sort of like things to do before our next visit. Sort of like things to help manage anxiety; homework to help manage my anxiety. (20, Female)*
	Nutrition *(Respondents described a preference for discussions about nutrition/healthy eating with their providers)*	*I came home with a food plate paper where it shows the amount of food that I should be eating and what my plate should look like with the carbs, the vegetables, what I should be drinking, how I should have fruits. (15, Female)*
	Administrative Needs *(Respondents described a preference for discussions about administrative needs, including forms and referrals)*	*After the visit when I came home, well she sent me home with my physical which is what we needed to send to the academy, which was the point of the appointment. (15, Female)*
	Topics not to Discuss *(Respondents reported topics they would like to discuss less with their providers)*	*None actually. There weren’t any things that I wanted to discuss less. I believe that at our meeting, at our appointment, we discussed all of the things that we needed to discuss. We didn’t discuss things less because they were discussed at the right amount that they had to be. (15, Female)*

## 4 Discussion and conclusion

### 4.1 Discussion

These findings revealed important preferences and priorities of adolescents around the information, education and materials received in the outpatient setting. Given the known gap in care delivery [[2], [3], [4], [5]], the satisfaction these adolescents had with the comprehensiveness of their visits and the high rate of AVS receipt, incorporating an AVS with timely and appropriate resources and information is critically important. While adolescents have a positive view of the AVS, there continues to be a gap in use of this effective tool [17]. Additionally, adolescent participants were focused on topics including mental health, nutrition and administrative needs. While this is in contrast to prior literature that has shown adolescents to be interested in a broad range of topics, though many studies focused on parental preferences [18,19], it is consistent with the current adolescent mental health crisis [23]. Highlighting these topics during the visit or in the AVS may help to further engage adolescent patients.

Adolescent preference for varied types of electronic and paper resources is consistent with previous literature emphasizing the need for multimodal methods of health information delivery [18,24]. With the increasing use of electronic and personally-curated resources [25], it is imperative providers compile accessible and reliable information for this population. This could be achieved through the use of tools such as the AVS and a focus on content deemed most important by patients, with each topic likely requiring specific communication avenues. By consistently providing adolescents with more targeted and patient-centered guidance, providers can expect to see greater retention of information [6], healthier behaviors and overall improved health outcomes. While the impact of the AVS on patient education and resources has been explored in other patient populations, there remains a need to improve understanding in adolescents. With a greater understanding of adolescent perceptions and needs, providers can give information to help improve adolescent engagement with their physicians and healthcare overall.

This study is limited as it is a single-institution and may not be generalizable to the broader adolescent population. However, it does include the perspectives of a predominantly Hispanic, low-income and publicly-insured group of adolescents, which is essential as this is a population with particularly low rates of annual preventive visits [3,26]. The response rate in our study was low, and exacerbated by parents not allowing their adolescent children to participate, and thus we may not have fully captured the perspectives of individuals who have less engagement with the healthcare system.

### 4.2 Innovation

Adolescents overall have low engagement with healthcare [[2], [3], [4], [5], [6]], yet there is a constant need to support their growing autonomy. Our results support a shift toward adolescent patient-centered care, as other improvement initiatives rarely apply adolescents’ self-reported experiences within healthcare [2]. This study demonstrates innovation as perceptions of educational materials e.g. the AVS, have been explored in other age groups, but not among adolescents. Given that adolescents themselves are the primary recipients of this education, their perspectives must be carefully considered.

Adolescent perspectives may help more effectively engage, educate and support patients by guiding the development of targeted instructions and resources. Next steps include determining the most impactful modes of information delivery, exploring the effectiveness of various elements of patient education in modifying adolescent behaviors, as well as comparing adolescent perceptions with those of their parents.

### 4.3 Conclusion

This qualitative study demonstrated positive perceptions of the AVS among adolescents in an urban ambulatory setting. Adolescent participants described their preference for multimodal delivery of educational material, specifically related to mental health and nutrition. Insights from adolescents can inform the development of future healthcare resources.

## Declaration of Competing Interest

The authors declare that they have no known competing financial interests or personal relationships that could have appeared to influence the work reported in this paper.
